# Mental Health During the First Year of the COVID-19 Pandemic: A Review and Recommendations for Moving Forward

**DOI:** 10.1177/17456916211029964

**Published:** 2022-01-19

**Authors:** Lara B. Aknin, Jan-Emmanuel De Neve, Elizabeth W. Dunn, Daisy E. Fancourt, Elkhonon Goldberg, John F. Helliwell, Sarah P. Jones, Elie Karam, Richard Layard, Sonja Lyubomirsky, Andrew Rzepa, Shekhar Saxena, Emily M. Thornton, Tyler J. VanderWeele, Ashley V. Whillans, Jamil Zaki, Ozge Karadag, Yanis Ben Amor

**Affiliations:** 1 Department of Psychology, Simon Fraser University; 2 Saïd Business School, University of Oxford; 3 Department of Psychology, University of British Columbia; 4 Institute of Epidemiology and Health, University College London; 5 Department of Neurology, New York University School of Medicine; 6 Vancouver School of Economics, University of British Columbia; 7 Department of Surgery and Cancer, Imperial College London; 8 Department of Psychiatry and Clinical Psychology, Saint George Hospital University Medical Center; 9 Centre for Economic Performance, London School of Economics and Political Science; 10 Department of Psychology, University of California, Riverside; 11 Global Food Security Program, Gallup Inc., London, England; 12 Department of Global Health and Population, Harvard T. H. Chan School of Public Health; 13 Department of Epidemiology and Biostatistics, Harvard T. H. Chan School of Public Health; 14 Negotiations, Organizations and Markets Unit, Harvard Business School; 15 Department of Psychology, Stanford University; 16 Center for Sustainable Development, Columbia University

**Keywords:** COVID-19, mental health, psychological distress, subjective well-being, loneliness, social connection, self-harm, suicide

## Abstract

COVID-19 has infected millions of people and upended the lives of most humans on the planet. Researchers from across the psychological sciences have sought to document and investigate the impact of COVID-19 in myriad ways, causing an explosion of research that is broad in scope, varied in methods, and challenging to consolidate. Because policy and practice aimed at helping people live healthier and happier lives requires insight from robust patterns of evidence, this article provides a rapid and thorough summary of high-quality studies available through early 2021 examining the mental-health consequences of living through the COVID-19 pandemic. Our review of the evidence indicates that anxiety, depression, and distress increased in the early months of the pandemic. Meanwhile, suicide rates, life satisfaction, and loneliness remained largely stable throughout the first year of the pandemic. In response to these insights, we present seven recommendations (one urgent, two short-term, and four ongoing) to support mental health during the pandemic and beyond.

The novel coronavirus (SARS-CoV-2) has infected millions of people and altered the lives of nearly every human on the planet. Although early fears focused on respiratory failure from contracting the virus, a fast-growing body of research points to the possibility that COVID-19 has a farther-reaching impact than originally recognized. Specifically, during the first year of the pandemic, most of the world’s population lived with the uncertainty of contracting the virus and with disruptions to daily life resulting from public-health measures implemented to slow the spread of COVID-19, which may have imposed psychological challenges. What are the mental-health consequences of living through a pandemic? What can individuals, organizations, and governments do to support mental health during this extraordinary time and beyond?

To help answer these questions, *The Lancet* assembled a COVID-19 commission to use evidence-based insights to understand the scope of the current pandemic and its consequences. This commission aims to “help speed up global, equitable, and lasting solutions to the pandemic” (Sachs et al., 2020). Part of this effort is captured in this article by the current authors, who represent the commission’s mental-health task force. Our aim is to summarize findings, delineate high-priority open questions, and offer recommendations for both individuals and organizations to support mental health during the current pandemic. We focus on mental health because it is a critical, consequential, and undersupported facet of overall well-being (Chisholm et al., 2016; Layard, 2014; Layard & Clark, 2015; Patel et al., 2018; World Health Organization, 1948). Indeed, mental illnesses affect between one third and one half of the working-age population in some countries, whereas only a small percentage of the population (~5%) receives access to evidence-based treatments that offer a favorable chance of recovery (Clark et al., 2018). Moreover, mental health holds personal, economic, and societal relevance given its greater impact on human activity than any other noncommunicable illness (Knapp & Wong, 2020) and association with higher rates of mortality (e.g., Chida & Steptoe, 2008; Howell et al., 2007; Keyes & Simoes, 2012; cf. Singer et al., 1976).

This article contains two sections. In the first section, we review the mental-health correlates of living through the pandemic using evidence collected through April 2021. In the second section, we offer seven recommendations (one urgent, two short-term, and four ongoing) and early insights for managing mental health during COVID-19 to *build back better.* (The White House, 2021) We call for urgent large-scale research into the nature, treatment, and long-term mental-health consequences of living through the pandemic (Recommendation 1). In the short term, we recommend more systematic monitoring of mental health for possible and confirmed patients as well as for people with higher exposure or burdens of care (Recommendation 2) and the prioritization of safe access to childcare and elementary schools (Recommendation 3). In the longer term, we encourage greater investment in mental-health services so that research-based treatment for mental health is as widely available as physical-health treatment (Recommendation 4). This includes making online mental-health therapy widely available and supplemented with in-person support (Recommendation 5); promoting widespread subjective well-being efforts at work, schools, and in communities (Recommendation 6); and embedding mental-health care and promotion within all social-care systems (Recommendation 7).

Before presenting the evidence, two limitations merit discussion. First, research on COVID-19 and its sequelae is evolving fast. Most of the evidence reviewed here was collected during the early months of the pandemic. Because the prevalence of the virus and public-health response patterns are in flux, the full picture is still unfolding. In an effort to present the most current and robust information, we focus on well-powered, representative, or weighted samples using rigorous methodologies (e.g., preregistration or well-matched control groups), including preprints that are currently undergoing peer review. We prioritized data with one or more of these qualities because such evidence is more likely to provide the most useful insights through robust estimates, generalizable conclusions, and nonspurious information. Second, much of the large-scale data available to date catalogues the impact of COVID-19 in relatively Western, educated, industrialized, rich, and democratic (WEIRD) nations (Henrich et al., 2010). For this reason, we are cautious about extrapolating these data to other nations and cultural contexts. More longitudinal and representative research is needed. However, we feel that several critical lessons have already emerged.

## What Are the Mental-Health Consequences of Living Through the Pandemic?

**Fig. 1.**
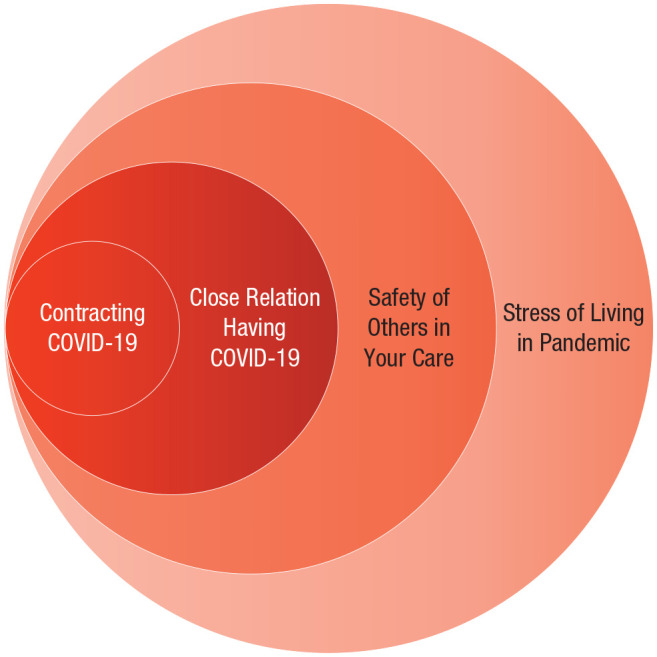
Stressors imposed on individuals by the COVID-19 pandemic. Each circle represents a layer of potential stress during the COVID-19 pandemic that may accumulate to undermine mental health.

Nearly everyone on the planet has been living through the pandemic and the public-health measures issued in response, which has imposed various stressors on individuals (see Fig. 1). How has the first year of the pandemic and the wide array of response actions affected mental health? To answer this question, we first introduce mental health as a broad, complex, and multifaceted construct that we consider through the lens of four key outcomes defined below. These outcomes were chosen through a bottom-up selection process in which our multidisciplinary team of experts surveyed the literature for high-quality research in the fall of 2020 and spring of 2021. The task-force members then met to discuss and evaluate the evidence, and we identified four outcomes that had been studied sufficiently to enable initial conclusions: psychological distress, self-harm, subjective well-being, and loneliness. These outcomes represent topics that have been widely assessed and discussed during the pandemic (e.g., Clay, 2020; Miller, 2020).

### Defining Our Constructs of Interest

*Psychological distress* captures a range of psychological states, including anxiety, depression, distress, and more (Kotov et al., 2017). Although many people experience low to moderate levels of these states in daily life, high levels can cause mental illness in which a person experiences severe and chronic disturbance to daily function and may be diagnosed and treated clinically by a mental-health professional (VandenBos, 2013). Here we focus on several representative constructs—mainly anxiety, depression, and distress—that have been measured in large samples with validated self-report screening and diagnostic tools, such as the Patient Health Questionnaire (PHQ-9; Kroenke et al., 2001), to assess depression. Many of these instruments allow researchers to compute a total score that can be compared with standard cutoffs to identify acute or severe levels.

*Self-harming behavior* is defined here as the deliberate, direct destruction or alteration of body tissue that results in damage (Gratz, 2001). We also consider suicidal ideation and suicide attempts. Although self-harming behavior is not considered a component of mental health, it is a maladaptive method of coping with overwhelming negative emotions that have harmful physical consequences and can interfere with interpersonal relationships and therapy (Favazza, 1989). Self-harm is typically measured using self-report tools, including the PHQ-9 (Kroenke et al., 2001), which asks participants about the frequency with which they have been “self-harming or deliberately hurting” themselves or have experienced “thoughts that you would be better off dead” in the previous week.

*Subjective well-being* is defined here as the extent to which someone reports experiencing a preponderance of positive affect or emotion and infrequent negative affect or emotion, as well as a positive evaluation of their life (Diener, 1984). Because positive and negative emotions tend to be relatively malleable and respond to changes in one’s immediate environment, emotions are typically assessed by asking people to indicate the frequency or extent to which they have recently felt positive states (e.g., happy, joyful, calm) and negative states (e.g., worried, sad, bored). In contrast, life-satisfaction ratings measure respondents’ assessment of their life as a whole or its facets (e.g., satisfaction with work or family life). For instance, one tool commonly used to measure life evaluations is the Cantril ladder (Cantril, 1965), which prompts respondents to rate their life on a ladder-like scale ranging from 0 at the bottom, reflecting the worst possible life, to 10 at the top, indicating the best possible life (Helliwell et al., 2019). As this measure suggests, life evaluations tend to be more cognitive in nature (Diener et al., 2003), and although such ratings may be informed by one’s current or recent emotions, life satisfaction tends to be more stable than emotion ratings. In this article, we focus on a variety of subjective well-being measures rather than objective well-being indicators.

*Loneliness* is a psychological state that is associated with deficiencies in a person’s social relationships. Although loneliness is not a facet of mental health per se, a wealth of research indicates that loneliness is a key predictor of mental-health challenges, such as distress (Luchetti et al., 2020; Perlman & Peplau, 1984). It is noteworthy that mental-health difficulties arise from the perceived discrepancy between one’s desired and actual social-relationship quality rather than merely being physically isolated (Holt-Lunstad, 2017). Loneliness is often assessed using self-report measures, such as the UCLA Loneliness Scale (J. Lee & Cagle, 2017), which captures individuals’ overall loneliness, as well as their feelings of isolation and the availability of social connections. In our review, we also assess the opposite of loneliness—social connection—captured on measures such as the Social Connectedness Scale (R. M. Lee et al., 2001), which focuses more on one’s degree of connection with others and less on feelings of isolation.

With these definitions in mind, how have mental health and these related constructs changed during COVID-19? Below we review the most informative data available to date to focus on three overarching questions (see Table 1). First, have average levels of psychological distress, self-harm, subjective well-being, and loneliness changed from prepandemic to during the pandemic? Second, what factors predict greater risk or protection in psychological distress, self-harm, subjective well-being, and loneliness during the pandemic onset and early months? Third, considering data collected after COVID-19 started, what are the correlates of better and worse mental health during the pandemic? Taken together, these questions offer a broad and useful summary of the fast-emerging literature on COVID-19 and mental health.

**Table 1.** Summary of the Repeated Cross-Sectional and Longitudinal Evidence Surveyed to Consider How Psychological Distress, Self-Harm, Subjective Well-Being, and Loneliness/Social Connection Have Been Affected by COVID-19

Evidence type and outcome	Study	Sample		Timing of data collection
		*N*	Location	
Psychological distress				
Cross-sectional				
Anxiety	Ebrahimi et al. (2021) ^a^	10,061	Norway^b^	Mar–Apr 2020
Depression	Ebrahimi et al. (2021) ^a^	10,061	Norway^b^	Mar–Apr 2020
Repeated cross-sectional				
Anxiety	Fujiwara et al. (2020)	1,982	U.K.^b^	Apr 2020
Depression	Ettman et al. (2020)	5,065	U.S.^b^	Mar–Apr 2020
Depression	Office for National Statistics (2021)	25,935	U.K.^b^	Jan–Mar 2021
Distress	McGinty et al. (2020)	1,468	U.S.^b^	Apr 2020
Longitudinal				
Anxiety	Fancourt et al. (2020)	36,520	U.K.	T1: Mar 2020; After: Weekly
Depression	Carr et al. (2020)	14 million clinical codes	U.K.	Jan 2019–Sept 2020
Depression	Fancourt et al. (2020)	36,520	U.K.	T1: Mar 2020; After: Weekly
Depression	Hensel et al. (2020)	108,075	International	Mar–Apr 2020
Distress	Banks & Xu (2020)	11,980	U.K.^b^	T1: 2010–2013; T2: 2014–2016; T3: 2017–2019; T4: Apr 2020
Distress	Daly & Robinson (2021)	7,319	U.S.^b^	Mar–July 2020
Distress	Pierce et al. (2020) ^a^	> 50,000	U.K.^b^	April 2020, ongoing
Distress	Proto & Quintana-Domeque (2021)	49,156	U.K.^b^	T1: 2017–2019; T2: Apr 2020
Self-harm				
Repeated cross-sectional				
Suicidal thoughts	Iob, Steptoe, et al. (2020)	44,774	U.K.	Mar–Apr 2020
Suicidal thoughts	Knudsen et al. (2021)	2,154	Norway	Jan–Sept 2020, Mar–May 2014–2018, 2020
Suicide	Pirkis et al. (2021)	21 countries		Apr–July 2020
Longitudinal				
Self-harm	Carr et al. (2020)	14 million clinical codes	U.K.	Apr–Sep 2020,
Suicidal thoughts	Brodeur et al. (2021) ^a^	Google trends		Jan 2019–Apr 2020
Suicide	Leske et al. (2020)	Australia		2015–2019, 2020
Subjective well-being				
Repeated cross-sectional				
Emotion	European Commission (2020)	30,000	Europe^b^	Sept–Dec 2020 and July–Aug 2020
Emotion	Fao et al. (2020)	99,719	U.K.^b^	June 2019–June 2020
Emotion	Helliwell et al. (2021)	~1,000/country in 95 countries	International^b^	2017–2019 and Mar–Dec 2020
Happiness	VanderWeele et al. (2020)	3,020	U.S.^b^	Jan 2020 and June 2020
Life satisfaction	Fujiwara et al. (2020)	1,982	U.K.^b^	Apr 2020
Life satisfaction	Helliwell et al. (2020)	49,200 and 4,200	Canada^b^	2018 and June 2020
Life satisfaction	Helliwell et al. (2021)	~1,000/country in 95 countries	International^b^	2017–2019 and Mar–Dec 2020
Life satisfaction	VanderWeele et al. (2020)	3,020	U.S.^b^	Jan 2020 and June 2020
Longitudinal				
Emotion	Brodeur et al. (2021) ^a^	Google trends		Jan 2019–Apr 2020
Emotion	Li et al. (2020)	17,865	China (Weibo users)	T1: Jan 13, 2020; T2: Jan 26, 2020
Emotion	Recchi et al. (2020)	779	France^b^	T1: 2017; T2: 2018; T3: Apr 1–8, 2020; T4: Apr 15–22, 2020; T5: Apr 29–May 6, 2020
Life satisfaction	Li et al. (2020)	17,865	China (Weibo users)	T1: Jan 13, 2020; T2: Jan 26, 2020
Life satisfaction	Liebig et al. (2020)	25,000	Germany	Apr 2020
Life satisfaction	University of Essex Institute for Social and Economic Research (2020)	34,318	U.K.^b^	T1: 2018–2019; T2: 2020–2021
Loneliness/social connection				
Repeated cross-sectional				
Loneliness	McGinty et al. (2020)	1,468	U.S.^b^	Apr/May 2018, Apr 2020
Loneliness	Tull et al. (2020)	500	U.S.	Mar–Apr 2020
Social connection	Tull et al. (2020)	500	U.S.	Mar–Apr 2020
Longitudinal				
Loneliness	Brodeur et al. (2021) ^a^	Google trends		Jan 2019–Apr 2020
Loneliness	Bu, Steptoe, & Fancourt (2020)	35,712	U.K.	T1: Mar 2020; After: Weekly
Loneliness	Fao et al. (2020)	99,719	U.K.^b^	June 2019–2020
Loneliness	Hansen et al. (2021)	10,740	Norway	T1: Oct 2019–Feb 2020 (varied by location); T2: June 2020
Loneliness	Luchetti et al. (2020) ^a^	1,545	U.S.	T1: Jan–Feb 2020; T2: Mar 2020; T3: Apr 2020
Social connection	Folk et al. (2020) ^a^	467; 336	Canada^c^; Mainly U.K. and U.S.	T1: Jan–Feb 2020; T2: Apr 2020

Note: Note: T1 = Time 1; T2 = Time 2; T3 = Time 3; T4 = Time 4.

a These studies were preregistered.

b These were nationally representative or probability-based samples.

c This was a college-student sample.

## Have Average Levels of Psychological Distress, Self-Harm, Subjective Well-Being, and Loneliness Changed From Before the Pandemic to During the Pandemic?

Headline: A clear and consistent body of evidence suggests that psychological distress increased during the early months of the COVID-19 pandemic and that most (but not all) facets returned to prepandemic levels by mid-2020. Although some components of subjective well-being showed signs of strain (e.g., increasing negative emotions), the data also revealed notable signs of resilience in life satisfaction, loneliness, social connection, and suicide.

To examine whether and how the COVID-19 pandemic has affected various facets and correlates of mental health, we draw on two types of evidence that offer unique strengths and insights: repeated cross-sectional data and longitudinal data. Repeated cross-sectional surveys compare responses from two or more data sets that are matched on key sample characteristics to examine whether and how average levels of mental-health outcomes have shifted over time. In contrast, longitudinal survey data assess responses from the same individuals over time, such as before and during the early months of the COVID-19 pandemic.

Considering repeated cross-sectional and longitudinal surveys in tandem is particularly informative because the two methodologies have many nonoverlapping strengths and weaknesses. For instance, because longitudinal surveys track the same people over time, this strategy minimizes concerns that different types of people were recruited for each survey and that individual differences (e.g., personality) might obscure relationships in the data. In addition, repeated cross-sectional data minimize worries that respondents who drop out of the study systematically differ from those who remain (i.e., selective retention) because each survey recruits a new but well-balanced sample. Although neither of these methodologies can confirm causality, consistent patterns observed across both types of evidence enable researchers to draw more convincing conclusions.

### Psychological distress

#### Repeated cross-sectional data

Numerous cross-sectional surveys suggest that the pandemic and its aftermath have taken a toll on various facets of psychological distress. For instance, within the United States, a comparison of two relatively large nationally representative surveys conducted before the pandemic (2017–2018; *n* = 1,441) and early on in the pandemic (March–April 2020; *n* = 5,065) shows a 3-fold increase in depression symptoms on the PHQ-9 (Ettman et al., 2020). Likewise, a nationally representative survey (*n* = 1,468) reported that 13.6% of American adults indicated symptoms consistent with severe psychological distress in April 2020, an estimate nearly 4 times greater than that observed in a separate nationally representative sample of Americans in 2018 (3.9%; *n* = 25,417; McGinty et al., 2020). Data from a nationally representative survey (*n* = 1,982) in the United Kingdom conducted in April 2020 also depicted significantly higher levels of anxiety than previous estimates from a similar, matched sample collected by the Office for National Statistics (ONS) between March and April 2019 (Fujiwara & Campbell, 2011; Fujiwara et al., 2020). A sample drawn primarily at random from adults in Norway (*n* = 10,061) between late March and early April 2020 showed an approximate 3-fold increase in the number of people surpassing the depression cutoff score of 10 on the PHQ-9 (30.78%) during the pandemic compared with a representative and random sample (10.24%; *n* = 1,944) drawn in 2015 (Ebrahimi et al., 2021). The same data set also suggest that the number of people surpassing the anxiety cutoff score of 8 on the Generalized Anxiety Disorder–7 was approximately twice as high during the pandemic (27.57%) compared with prepandemic estimates from nearby nations, such as Sweden (14.70%; *n* = 3,001; Johansson et al., 2013).

#### Longitudinal data

Pierce and colleagues (2020) found evidence of increased psychological distress during COVID-19, consistent with the cross-sectional data reported above. Using data from the U.K. Household Longitudinal Survey, which includes responses from more than 50,000 residents across England, Wales, Scotland, and Northern Ireland, the authors looked for changes in psychological distress on the General Health Questionnaire (GHQ) captured during COVID-19 (April 2020) to those collected before. Pierce and colleagues (2020) reported that the prevalence of clinically significant distress (i.e., the percentage of people surpassing clinical thresholds) rose from 18.9% in 2018 and 2019 to 27.3% in late April 2020 during lockdown among the full sample. In addition, the sample reported higher mean levels of psychological distress on the GHQ (an overall increase in scores from 11.5 to 12.6) during the same time frame, a gap that is nearly half a point larger than would be expected given the upward trend in psychological distress observed over the past few years (Pierce et al., 2020; see also Banks & Xu, 2020; Proto & Quintana-Domeque, 2021).

Over time, mean levels of psychological distress may have declined from an early peak after the COVID-19 outbreak. Supporting this possibility, longitudinal data from a nationally representative sample of 7,319 Americans in an eight-wave survey conducted between March and July 2020 revealed a significant increase in psychological distress between March and April but a return to prepandemic levels by June 2020 (Daly & Robinson, 2021). Likewise, responses from a large panel survey in the United Kingdom (*n* > 70,000) revealed that depression and anxiety, which were very high in March 2020, decreased precipitously in the first few weeks of lockdown and then plateaued (Fancourt et al., 2020). These findings align with those of Hensel et al. (2020), who reported that a nationwide lockdown in the United Kingdom was associated with lower levels of depression and worry in a large online sample (*n* = 108,075) collected in March and April 2020. A similar conclusion comes from a meta-analysis of 65 longitudinal cohort studies published between January 2020 and January 2021 (*n* = ~55,000 participants) tracking psychological distress from before to during the pandemic. The meta-analysis reported a significant increase in both anxiety and depression from March to April 2020 but then a decline to near prepandemic levels on most measures except depression by mid-2020 (Robinson et al., 2022). Consistent with this summary, the most recent analysis of the Opinions and Life Survey collected from a population-weighted cross-sectional sample of nearly 26,000 adults in Britain suggests that depression was still elevated in early 2021. Specifically, between late January and early March 2021, approximately 21% of the population reported a score of 10 or higher on the PHQ-9, which is more than double the prepandemic estimate of 10% captured between July 2019 and March 2020 (ONS, 2021).

As another approach to examining distress, researchers compiled patient records from more than 14 million individuals in the United Kingdom. They found that rates of mental illness were lower in April 2020 than expected on the basis of past trends but largely returned to expected levels by September 2020 (Carr et al., 2021). These initial declines may have been due to reductions in primary-care visits and fewer new diagnoses as a result of limitations caused by the COVID-19 pandemic. Thus, these data suggest an increase in unmet needs that could trigger a later influx in mental-health care (Carr et al., 2021).

### Self-harm

#### Repeated cross-sectional data

During the early months of the pandemic, thoughts of self-harm and suicide increased in the United Kingdom but not in Norway. Data from a population-weighted sample of 44,775 adults in the United Kingdom contacted between late March and late April 2020 indicate that 18% of respondents had thoughts about suicide or self-harm (Iob, Steptoe, et al., 2020). This estimate is higher than one captured in a previous survey by McManus et al. (2016) that showed that approximately 5.4% of the adult population 16 years and older from England, Scotland, and Wales had reported suicidal thoughts in the past year. However, responses to a diagnostic psychiatric interview completed by four waves of probability-based samples in Norway (*n* = 2,154) through January to September 2020 show no change in suicidal ideation over time (Knudsen et al., 2021).

#### Longitudinal data

A large-scale longitudinal data set indicates little change or an initial decline in self-harming behavior during the early months of the COVID-19 pandemic. Researchers analyzing more than 14 million patient records in the United Kingdom (also referenced above) found that self-harm rates were lower than expected in April 2020 and reverted to expected levels in many areas by September 2020 (Carr et al., 2021). Once again, however, lower levels of self-harm ratings may result from lower levels of detection and primary-care visits during the COVID-19 pandemic. Thus, it is possible that earlier unmet needs may require greater care in the future (e.g., Carr et al., 2021).

Critically, several sources demonstrate little change in suicide during the early months of the pandemic. Google trends spanning from January 2019 to April 2020 show decreases in searches referring to suicide in Western European countries and the United States (Brodeur et al., 2021). Real-time data from police reports in Queensland, Australia, show that suicide rates did not increase during the first 7 months of the COVID-19 pandemic compared with averages from 5 years prior (Leske et al., 2020). Researchers found no evidence for an increase in suicides in the Norwegian Cause of Death Registry from March to May 2014 through 2018 and March to May 2020 (Knudsen et al., 2021). Finally, researchers analyzing real-time suicide data from official government sources in 21 countries (16 high-income and five middle-income) using interrupted time-series analyses found no evidence of increased suicide from April 1 to July 30, 2020, in a comparison of observed and expected rates (Pirkis et al., 2021). Indeed, suicide rates were significantly *lower* than model expectations in some countries and regions (e.g., Chile, Ecuador, Japan, the United States) during this time frame. This pattern of results remained largely unchanged when including data up to October 31, 2020, where available, but two places did show a significant increase in October 2020 (Japan and Puerto Rico; Pirkis et al., 2021).

### Subjective well-being

#### Repeated cross-sectional data

Evidence suggests that people have experienced more unpleasant emotions during the pandemic. For example, comparing responses from approximately 1,000 people drawn from each of the 95 countries surveyed by the Gallup World Poll from March to December 2020 to average responses from 2017 to 2019 showed small but statistically significant increases in the frequency of negative emotions (rising from 27% to 29%), although there was no change in the frequency of positive emotions (Helliwell et al., 2021). Likewise, in April 2020, a nationally representative sample from the United Kingdom (*n* = 1,982) reported lower levels of daily happiness than reported by a previous sample in March and April 2019 (Fujiwara et al., 2020). Fao and colleagues (2020) analyzed responses from repeated cross-sectional panels collected by YouGov from June 2019 to June 2020 consisting of approximately 2,000 people per week (*n* = 99,719) representative in age, gender, social class, and education of the United Kingdom. These data revealed a sharp increase in negative affect and decrease in positive affect during the spring of 2020, with a partial return to typical levels by May 2020. A similar pattern emerged in data from the U.K. Understanding Society survey (University of Essex Institute for Social and Economic Research, 2020). Finally, a comparison of two separate nationally representative samples of Americans surveyed in January (*n* = 1,010) and June 2020 (*n* = 3,020) suggests that people’s feelings of happiness declined by almost 1 point on an 11-point scale (VanderWeele et al., 2020).

Although emotional measures of happiness exhibited declines during the early phase of the pandemic, life satisfaction (the cognitive-evaluative component of subjective well-being) remained largely unchanged in many countries. For example, data from the summer 2020 edition of the Eurobarometer, which sampled more than 30,000 people in 34 countries before (September–December 2019) and during (July–August 2020) COVID-19, showed very small changes, with more being positive than negative (European Commission, 2020). In the Gallup World Poll data (referred to above), life satisfaction exhibited a slight but nonsignificant *increase* from the pooled 2017–2019 averages compared with the corresponding 2020 data (Helliwell et al., 2021). However, life satisfaction appears to have declined in some countries. In Canada, life satisfaction dropped by 1.38 points on a 0 to 10 response scale, from 8.09 in 2018 (*n* > 49,200) to 6.71 in June 2020 (*n* = ~4,200), as measured in two nationally representative surveys (Helliwell et al., 2020). Likewise, nationally representative samples exhibited reduced life satisfaction in the United States (VanderWeele et al., 2020) and the United Kingdom (Fujiwara et al., 2020). The finding that so many other countries exhibited striking resilience requires further analysis. One explanation is that life evaluations invite people to compare their life to the lives of others. In doing so, people may feel that their lives are worse now than before in some respects but, on balance, are much better than they might have been.

#### Longitudinal data

Few longitudinal studies capture subjective well-being before and after COVID-19, but these studies depict a high degree of resilience. One data set included an assessment of positive and negative emotions among a probability sample of 779 individuals in France before (2017, 2018) and during (early April 2020, mid-April 2020, and late April/early May 2020) COVID-19. Overall, the data revealed an *increase* in emotional well-being over time, from before COVID-19 (mean of .64 in 2019) to during the pandemic (mean of .69 in May 2020; Recchi et al., 2020; see also http://www.cepremap.fr/Tableau_de_Bord_Bien-Etre.html). Data from the German Socio-Economic Panel, which includes a longitudinal sample of about 25,000 respondents in 15,000 households annually, showed that overall life satisfaction remained unchanged from 2019 to April 2020 (Liebig et al., 2020).

Looking beyond self-report scales, social-media posts and Internet searches can provide some additional insight into subjective well-being trends. A sentiment analysis of 17,865 active users of Weibo, China’s most popular social-media platform, spanning a 2-week period from January 13 to January 26, 2020 (with the COVID-19 outbreak declared a type B infectious disease by the National Health Commission on January 20), revealed a small but significant increase in negative emotion, depression, and indignation alongside decreases in positive emotion, including happiness and life satisfaction (Li et al., 2020). Meanwhile, Google searches for contentment, sadness, and irritability did not change from January 2019 to April 2020 in Western Europe and increased in the United States, but well-being searches over this same period declined in Western Europe and increased in the United States (Brodeur et al., 2021).

### Loneliness and social connection

#### Repeated cross-sectional data

Evidence concerning loneliness and social connection depicts some evidence of resilience as well. Despite early speculation and fear that physical distancing would unleash a second epidemic of loneliness, repeated cross-sectional studies have found little evidence of substantial change. For instance, responses to the weekly representative U.K. cross-sectional panel collected by YouGov described above from June 2019 to June 2020 (*n* = 99,719) suggests that the percentage of people reporting feeling lonely in the week before reporting peaked during the first month of lockdown from late March to April 2020, but rates began to decline in May and were within 2% of prepandemic levels at approximately 16% by June 2020 (~18%; Fao et al., 2020). Likewise, McGinty and colleagues (2020) examined data from a nationally representative sample of 1,468 Americans in April 2020 and found that 13.8% reported feeling that they were often or always lonely. This percentage is only slightly (albeit significantly) higher than the 11% reported in a separate sample in April and May 2018 (DiJulio et al., 2018). As a result, McGinty and colleagues (2020) suggest that loneliness is unlikely to be the primary source of distress during the COVID-19 pandemic.

#### Longitudinal data

Consistent with the repeated cross-sectional data reported above, longitudinal data flanking the pandemic show relatively little overall change in social connection and loneliness. For instance, responses from a nationwide sample of 1,545 Americans surveyed in late January/early February, March, and April 2020 exhibited no mean-level change in loneliness and reported a significant *increase* in perceptions of social support, although people with higher levels of loneliness at baseline were more likely to drop out of the study (Luchetti et al., 2020). Google search trend data align with these findings. Queries for loneliness did not increase significantly between January 2019 and April 2020 in the United States but did increase significantly during the first few weeks of lockdown in Western Europe before returning to baseline (Brodeur et al., 2021). In a preregistered, longitudinal study, Folk and colleagues (2020) found relatively little change in social connection before and during the pandemic among students in Canada (*n* = 467) and adults primarily in the United States and United Kingdom (*n* = 336). As Folk and colleagues noted, this resilience aligns with the idea of *substitution*, in which people find creative ways to fulfill their fundamental need to belong when familiar channels are blocked (Baumeister & Leary, 1995). Finally, a longitudinal data set including responses from 10,740 Norwegians contacted before the COVID-19 pandemic (in either October 2019 or February 2020) and again during the pandemic in June 2020 showed that overall loneliness remained stable or declined (Hansen et al., 2021).

Data collected after the onset of COVID-19 are consistent with the idea that loneliness has remained largely stable. A large (*n* = 35,712) longitudinal data set in the United Kingdom showed no mean-level change in loneliness over late March to early May 2020, although individuals with the highest levels of loneliness at baseline did become more lonely over time (Bu, Steptoe, & Fancourt, 2020). Likewise, an online sample of 500 Americans surveyed in late March and early April 2020 revealed that individuals who perceived the greatest impact of COVID-19 were most likely to report *lower* levels of loneliness and the *highest* levels of social support (Tull et al., 2020). Thus, the physical-distancing requirements of the COVID-19 pandemic may have encouraged many people to find new, creative forms of social connection. However, it is worth acknowledging that these studies examining social connection and loneliness during COVID-19 used online-recruitment and survey tools that required participants to possess at least a basic level of digital literacy to respond. Thus, this group of respondents may have had higher social-connection ratings because they were likely better able to remain connected to friends and family online compared with individuals who struggled with digital literacy and access to digital technologies.

## What Factors Predict Greater Risk or Protection in Psychological Distress, Self-Harm, Subjective Well-Being, and Loneliness During the Pandemic Onset and Progression?

Headline: Many preexisting inequalities in psychological distress remain. The pandemic has also introduced new profiles of risk, with younger individuals, females, and those with children under the age of 5 years showing the largest increase in psychological distress.

Appreciating the large-scale and far-reaching influence of COVID-19 on mental health is valuable, but mean-level changes can mask significant variation in who has been affected the most. Below, we consider how COVID-19 may have affected psychological distress, self-harm, subjective well-being, and loneliness/social connection above and beyond preexisting discrepancies in mental health.

To do so, we consider longitudinal data sets that assessed psychological distress, self-harm, subjective well-being, and loneliness/social connection in the same individuals before and during the early months of COVID-19. These data provide insight into whether and how various factors (e.g., personality, socioeconomic status, relationship status, mental-health history, family composition) predicted changes in mental health and related constructs as the pandemic substantially altered daily life. These data offer researchers the opportunity to examine whether some predictors remained the same and whether new predictors have emerged.

### Psychological distress

Some of the most rigorous evidence collected to date reveals that many of the preexisting risk factors for psychological distress have persisted during the COVID-19 pandemic, and several new profiles of risk have emerged. As noted above, Pierce and colleagues (2020) used longitudinal data from before and during the early months of COVID-19 (April 2020) from more than 50,000 individuals in the United Kingdom to assess mental-health changes. Although the data revealed an increase in psychological distress, they also showed that several previously documented predictors of psychological distress remain during the pandemic. Specifically, individuals who self-identify into the following categories report higher psychological distress under COVID-19: female (vs. male), member of a minority or marginalized racial group (e.g., Asian vs. White British), living in urban (vs. rural) areas, those in the lowest income quintile (vs. other income quintiles), unemployed or inactive (vs. employed), living without a partner (vs. living with a partner), or having preexisting health risks (vs. not; Pierce et al., 2020). Many of these factors were robust predictors of psychological distress before the pandemic, indicating that preexisting mental-health divides remain under COVID-19.

Critically, individuals in some groups have suffered more during the pandemic than before, introducing new profiles of risk. Within-individual analyses controlling for time trends and other sources of change suggest that COVID-19 led to the greatest increases in psychological distress for individuals who identify as female, are in younger age categories (18–24 and 25–34), and have young children (< 5 years old) at home (Pierce et al., 2020). These findings are consistent with those of Banks and Xu (2020), who analyzed responses from 11,988 individuals in the same data set (U.K. Household Longitudinal Survey) and reported the greatest declines in mental health among young women (see also Proto & Quintana-Domeque, 2021).

Pierce and colleagues (2020) also indicated that individuals who were employed or retired before the pandemic reported higher than expected levels of psychological distress in April 2020, as did individuals in the lowest and highest income brackets. This latter finding is somewhat surprising given that longitudinal responses from 12,527 adults in the United Kingdom collected between late March to mid-April 2020 show that COVID-19 adversities have disproportionately affected people in lower socioeconomic groups (Wright et al., 2020). However, most of these findings align with other data indicating that individuals who are younger (Imperial College London/YouGov Tracker; Varma et al., 2020), female (Fancourt et al., 2020), and experiencing financial strain (Varma et al., 2020) report higher psychological distress during the pandemic. Thus, the pandemic has maintained the impact of some (but not all) risk factors for psychological distress and introduced new risk profiles as well (e.g., those who are young, female, and with young children at home).

### Self-harm

Several large-scale data sets now suggest that suicide rates have not increased above predicted rates during the first several months of the pandemic (e.g., Pirkis et al., 2021). However, we are aware of only one article examining how suicide patterns have changed for various demographic and occupational groups over time using data from Japan (Ueda et al., 2021). These data note an initial decline in suicides between April and May 2020 but a rise in July 2020 and afterward. The authors examined changes in suicide rates by gender, age, and occupation status and observed the largest changes among women under the age of 40. For instance, in October 2020, suicides were nearly 96% higher among young women than the average number of suicides during the month of October in 2017, 2018, and 2019 for this group. Suicides were also higher among students and homemakers in 2020 than in 2017, 2018, and 2019. Thus, these data dovetail with those reported above in that they suggest that some of the increases have been most extreme among young women.

### Subjective well-being

As reported above, a longitudinal data set of 779 people in France showed an overall increase in emotional well-being from before to during (May 2020) the pandemic (Recchi et al., 2020; see also http://www.cepremap.fr/Tableau_de_Bord_Bien-Etre.html). These changes appear to be similar across most subgroups of the population. Indeed, whereas individuals in the lowest income bracket reported significantly lower levels of subjective well-being than individuals in middle and high income brackets before the pandemic (Howell et al., 2006; Kahneman et al., 2006; Reyes-García et al., 2016), the pandemic did not alter this pattern. Subjective well-being gains appear to be relatively uniform for individuals across the income spectrum and with different occupations (Recchi et al., 2020). Recchi and colleagues (2020) propose that such similar increases in subjective well-being reported in this sample may be due to France’s generous unemployment benefits paid to full-time employees during the pandemic (Recchi et al., 2020). This possibility is consistent with an analysis of past quarantine measures and their impact on subjective well-being wherein various forms of governmental aid offered useful support during a challenging time (Brooks et al., 2020).

### Loneliness and social connection

As noted above, longitudinal data flanking the start of the pandemic show relatively little overall change in loneliness and social connection. The researchers examining responses from a nationwide sample of 1,545 Americans surveyed in late January/early February, March, and April 2020 collected information on respondents’ age, health status, and living arrangements (alone vs. two or more people in a household). At baseline, people in younger age categories, living alone, and those experiencing one or more chronic health concerns reported greater loneliness. However, only age predicted greater *changes* in loneliness during the onset of COVID-19, such that older adults reported greater increases in loneliness between late January/early February and March. Loneliness changes across age groups were similar between March and April. Furthermore, the data suggest that COVID-19 did not differentially affect loneliness for individuals with varying health statuses or various living arrangements (Luchetti et al., 2020).

Other data align with the general pattern of stability in loneliness and offer some insight into protective factors. As noted above, Hansen and colleagues (2021) found no overall change in loneliness among a longitudinal sample of 10,740 Norwegian adults contacted several months before (October 2019 or February 2020) and during (June 2020) the COVID-19 pandemic. However, subsequent analyses revealed that having a romantic partner, being younger in age (< 65 years, among women), as well as having lower social support and higher psychological distress predicted greater decreases in loneliness over time. The potential importance of living with a partner has been observed in other longitudinal samples utilizing preregistered analysis plans (e.g., Okabe-Miyamoto et al., 2021), suggesting that living with a romantic partner during this challenging time may offer unique benefits.

## Looking at Data Collected After COVID-19 Started, What Experiences and Behaviors Are Associated With Higher or Lower Psychological Distress, Self-Harm, Subjective Well-Being, and Loneliness During the Pandemic?

Headline: Being near or experiencing COVID-19 infection, struggling with financial uncertainty introduced by COVID-19, and spending more time homeschooling, engaged in chores, or reading COVID-19 news has been associated with more psychological distress and worse subjective well-being.

Data collected in the wake of COVID-19 shed light on several factors associated with psychological distress, self-harm, subjective well-being, and loneliness during the pandemic. For simplicity and brevity, we discuss findings on our four key outcomes (psychological distress, self-harm, subjective well-being, and loneliness/social connection) under broad category headings here. A more detailed list of study details can be found in Table 2.

**Table 2.** List of Studies Discussed Probing the Impact of Personal Experience, Financial Hardship, and Time Use on Psychological Distress, Self-Harm, Subjective Well-Being, and Loneliness/Social Connection During COVID-19

Outcome and study	Sample		Timing of data collection	Nature of the data
	*N*	Location		
Personal experience				
Anxiety				
Cao et al. (2020)	7,143	China^a^	Jan or Feb 2020	Cross-sectional
Li et al. (2020)	17,865	China (Weibo users)	T1: Jan 13, 2020; T2: Jan 26, 2020	Longitudinal
Planchuelo-Gómez et al. (2020)	1,056	Spain	T1: Mar/Apr 2020; T2: Apr/May 2020	Longitudinal
Wathelet et al. (2020)	69,054	France^a^	Apr–May 2020	Cross-sectional
Depression				
Iob, Frank, et al. (2020)	51,417	U.K.	Mar–Apr 2020	Longitudinal
Li et al. (2020)	17,865	China (Weibo users)	T1: Jan 13, 2020; T2: Jan 26, 2020	Longitudinal
Wathelet et al. (2020)	69,054	France^a^	Apr–Mar 2020	Cross-sectional
Distress				
Pierce et al. (2020)^b^	>50,000	U.K.^c^	Apr 2020	Cross-sectional
Suicidal thoughts				
Iob, Steptoe, et al. (2020)	44,774	U.K.	Mar–Apr 2020	Cross-sectional
Wathelet et al. (2020)	69,054	France^a^	Apr–May 2020	Cross-sectional
Financial hardship				
Anxiety				
Cao et al. (2020)	7,143	China^a^	Jan or Feb 2020	Cross-sectional
Wathelet et al. (2020)	69,054	France^a^	Apr–May 2020	Cross-sectional
Depression				
Wathelet et al. (2020)	69,054	France^a^	Apr–May 2020	Cross-sectional
Suicidal thoughts				
Wathelet et al. (2020)	69,054	France^a^	Apr–May 2020	Cross-sectional
Time use				
Anxiety				
Bu, Steptoe, Mak, & Fancourt (2020)	35,712	U.K.	T1: Mar 2020; After: Weekly	Longitudinal
Gao et al. (2020)	4,872	China	Jan–Feb 2020	Cross-sectional
Huckins et al. (2020)	178	U.S.^a^	T1: Aug–Nov 2018; T2: Jan 2020	Longitudinal
Planchuelo-Gómez et al. (2020)	1,056	Spain	T1: Mar/Apr 2020; T2: Apr/May 2020	Longitudinal
Wathelet et al. (2020)	69,054	France^a^	Apr–May 2020	Cross-sectional
Depression				
Bu, Steptoe, Mak, & Fancourt (2020)	35,712	U.K.	T1: Mar 2020; After: Weekly	Longitudinal
Ebrahimi et al. (2021)^b^	10,061	Norway	Mar–Apr 2020	Cross-sectional
Huckins et al. (2020)	178	U.S.^a^	T1: Aug–Nov 2018; T2: Jan 2020	Longitudinal
Martinez et al. (2020)	1,613	Brazil	May 2020	Cross-sectional
Wathelet et al., 2020	69,054	France^a^	Apr–May 2020	Cross-sectional
Happiness				
Giurge et al. (2021)^b^	30,018	International	Mar–June 2020	Longitudinal
Negative affect				
Lades et al. (2020)	604	Ireland	Mar 2020	Cross-sectional

Note: T1 = Time 1; T2 = Time 2; T3 = Time 3; T4 = Time 4.

a This was a college-student sample.

b These studies were preregistered.

c These were nationally representative or probability-based samples.

### Personal experience with or proximity to illness

Believing that you or a close other has contracted COVID-19 is associated with psychological distress and self-harm. Examining data from 44,775 people surveyed between late March to late April in the United Kingdom, Iob, Steptoe, et al. (2020) found that individuals who had received a diagnosis of COVID-19 reported higher levels of self-harm and suicidal thoughts than those who had not. Even those without confirmation of the virus reported mental-health costs. For instance, in a sample of 69,054 quarantined college students in France surveyed between April and May 2020, students reported greater distress if they experienced symptoms consistent with COVID-19 (Wathelet et al., 2020), suggesting that simply worrying that one has the virus may lead to psychological distress (see Fig. 1). However, the correlational nature of these data allows for the possibility that individuals with greater mental concerns may be more likely to be hypervigilant and distressed by virus symptoms. Beyond personal risk, concern about close others contracting COVID-19 is also associated with psychological distress (see Fig. 1). For instance, in a sample of 7,143 college students surveyed in China in January and February 2020, people who reported that their friends or family had been infected reported higher anxiety (Cao et al., 2020; see also Li et al., 2020; Wathelet et al., 2020).

Along similar lines, health-care workers who treat numerous patients with COVID-19 and see the fatally ill in large numbers may also report greater psychological distress (Gruber et al., 2021; Vigo et al., 2020; Fig. 1). Providing some support for this possibility, data from the U.K. Household Longitudinal Survey (*n* > 50,000) collected in April 2020 revealed that health-care workers were more likely to report psychological distress scores surpassing clinical thresholds than non-health-care workers, but mean-level rates did not differ from those working outside the health-care industry and did not increase significantly more than those of non-health-care workers during the early months of COVID-19 (Pierce et al., 2020; see also Iob, Frank, et al., 2020). Longitudinal data collected from 1,056 adults in Spain in March 2020 and April and May 2020 show that health-care workers reported higher anxiety scores than non-health-care workers at the early peak of deaths (Planchuelo-Gómez et al., 2020). Responses from this same sample also indicate that anxiety dropped significantly among health-care workers (vs. non-health-care workers) 1 month later when the number of deaths decreased (Planchuelo-Gómez et al., 2020). As infection rates in second and subsequent waves rise in many countries, the psychological distress experienced by those with personal experience or connections to COVID-19 are expected to grow. Indeed, a rapid expert consultation of health-care workers from December 2020 noted that the full scope of the mental-health impact on health-care workers remains to be seen (National Academies of Sciences, Engineering, and Medicine [NEM], 2020). However, health-care workers were also at greater risk of psychological distress before the pandemic, and insight from previous outbreaks (SARS) warns that stress, insomnia, and suicide could follow (NEM, 2020).

### Economic hardship

Because financial resources provide basic needs (e.g., safe housing, food) for oneself and one’s family, self-reported financial strain imposed by the pandemic is associated with psychological distress. Indeed, between April and May 2020, French college students (*n* = 69,054) who indicated that they had experienced a greater loss in income reported higher anxiety, distress, stress, depression, and suicidal ideation than those who did not (Wathelet et al., 2020). Likewise, greater concern about the economic impact of the pandemic predicted higher levels of anxiety among a sample of 7,143 college students in China surveyed during January or February 2020 (Cao et al., 2020).

### Time use

Several large-scale data sets offer insight into what types of behaviors are associated with higher or lower psychological distress and subjective well-being during the pandemic. For instance, in data from a longitudinal panel of 55,024 individuals in the United Kingdom surveyed between late March to late May 2020, within-person increases in time spent gardening or time in nature, exercising, reading, or listening to music predicted decreases in depression (Bu, Steptoe, Mak, & Fancourt, 2020; see also Ebrahimi et al., 2021; Martinez et al., 2020). Likewise, on days when people spent more time gardening, they felt reduced levels of anxiety, and on days when people spent more time volunteering, gardening, or exercising, they reported greater life satisfaction (Bu, Steptoe, Mak, & Fancourt, 2020). Meanwhile, more time spent following COVID-19 news predicted higher depression, higher anxiety, and lower life satisfaction in numerous data sets (Bu, Steptoe, Mak, & Fancourt, 2020; Gao et al., 2020; Huckins et al., 2020; Lades et al., 2020; Planchuelo-Gómez et al., 2020; Wathelet et al., 2020).

Several data sets display a negative association between time spent engaging in childcare or homeschooling and subjective well-being as well as greater psychological distress. For instance, longitudinal data from the same 55,024 individuals in the United Kingdom contacted in late March to late May 2020 showed that more time spent in childcare was associated with increased feelings of depression and lower life satisfaction (Bu, Steptoe, Mak, & Fancourt, 2020; Lades et al., 2020). Although prepandemic data from 909 working women in the United States suggest that childcare is not a particularly enjoyable activity (Kahneman et al., 2004), the pandemic forced school and daycare closures around the world, which has caused a dramatic increase in childcare demands for many parents. Data from 16,908 adults in the United Kingdom and United States surveyed between March and April 2020 indicate that women are spending more time in these caregiving roles and doing household chores (Adams-Prassl et al., 2020). Thus, it is not surprising that pooled data from 31,141 adults in several countries (e.g., Brazil, Canada, Denmark, the United States) contacted between March and June 2020 found that women engage in greater caretaking and household chores, with the latter predicting lower happiness (Giurge et al., 2021).

### Summary

Taken together, evidence collected during the first year of the pandemic points to several conclusions. First, repeated cross-sectional and longitudinal data sets converge to document a significant rise in psychological distress during the early months of the pandemic, which was especially pronounced among individuals who are young, female, and parents to children under 5 years of age. However, several sources of data suggest that most (but not all) metrics of psychological distress returned to baseline, on average, by mid-2020. Second, numerous sources showed no increase in suicide rates across more than 20 nations. Third, nationally representative data sets depicted little change, if any, in life satisfaction across most countries, with notable exceptions (e.g., Canada, United Kingdom, the United States). Likewise, several data sets document little to no change in loneliness. Finally, evidence suggests that individuals with closer proximity to illness, higher economic strain, and more household chores and childcare are at greater mental-health risk. Meanwhile, people report experiencing greater mental health on days when they exercise, spend time in nature, read, or volunteer during the COVID-19 pandemic.

## Recommendations

**Fig. 2.**
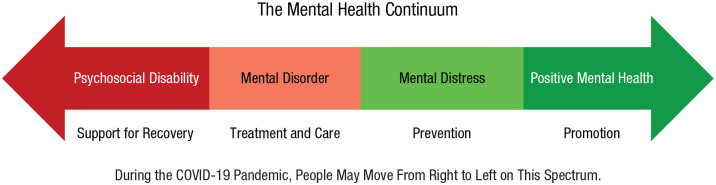
The mental-health continuum.

The data above describe the varied, unequal, and complex changes in mental health observed in the face of the COVID-19 pandemic. Although we have tried to synthesize the most informative studies to convey robust patterns of evidence, knowing how to respond to these insights may be unclear. Therefore, to help governments, businesses, and individuals take action, we offer seven research-grounded recommendations (one urgent, two short-term, and four ongoing) for supporting mental health during the pandemic and beyond (Table 3). These recommendations aim to help people across the spectrum, from mental illness to well-being (see Fig. 2).

**Table 3.** Summary of Recommendations for Addressing and Supporting Mental Health During the COVID-19 Pandemic and Beyond

Urgent	
1	Support immediate, large-scale research into the nature, treatment, and long-term consequences of COVID-19 on mental health.
Short-term	
2	Encourage physicians, nurses, and other mental health care professions to systematically screen for and monitor a range of short- and long-term mental health dimensions among COVID-19 survivors, close relations, as well those with greater exposure risk or burden of care.
3	Prioritize safe access to childcare and elementary schooling.
Ongoing	
4	Invest in mental health care such that someone with mental illness has equal access to evidence-based treatment as someone who has physical illness.
5	Specific mental health resources and actions should be tailored to the resources available, but at the very least should include online cognitive behavior therapy treatments supplemented by locally trained, although possibly lay, mental health practitioners.
6	Individuals and organizations should supplement existing mental health care with well-being promotion.
7	Governments and organizations should facilitate access to mental health care and the promotion of well-being alongside social care.

### Urgent recommendations

*Recommendation 1.* We call on researchers, governments, and funding bodies to support immediate, large-scale research to understand the nature, treatment, and long-term consequences of COVID-19 on mental health and the brain (Holmes et al., 2020). Indeed, although this article summarizes the rapidly growing and evolving evidence on COVID-19 and mental health, we do not yet know the duration and long-term impacts of this global challenge. As the number of people infected with the virus continues to climb, humanity needs greater insight into how to support those who become infected, as well as those who care for the infected (de Erausquin et al., 2020). Moreover, greater knowledge is needed to understand how most people have altered their lives, as well as what factors have supported or challenged mental health during this time. Future challenges (pandemic or otherwise) lie ahead. Increased psychological insight from this unprecedented event can help inform decision-making and policy.

### Short-term recommendations

*Recommendation 2.* We encourage physicians, nurses, and other mental-health professionals to systematically screen for and monitor a range of short- and long-term mental-health dimensions among COVID-19 survivors and close relations. Given the evidence above indicating that various forms of personal experience with the virus—from worries about personal safety to concern that close others have contracted COVID-19 (e.g., Wathelet et al., 2020)—are associated with greater psychological distress, public-health nurses or volunteers could follow up with patients, COVID-19 test takers, and family of those who have been ill as a result of COVID-19 to screen for psychological-distress concerns. Early awareness of distress could trigger contact from a trained professional, who could then direct the individual to local mental-health resources. Likewise, just as people with greater burden of care (e.g., health-care workers, teachers) and risk exposure (e.g., grocery-store clerks, factory workers) have been prioritized for physical care through early vaccination, so too should these individuals be monitored and supported with greater mental-health care (see Fig. 1).

*Recommendation 3.* We recommend prioritizing safe access to childcare and elementary schooling during the pandemic. Early education and childcare provide learning, socialization, and food-access opportunities to countless children around the world, as well as intervention for safety when needed (e.g., Hoffman & Miller, 2020; Mayurasakorn et al., 2020). In addition, elementary education and childcare allows working parents to attend to employment tasks with fewer disruptions, chores, and multitasking demands. Thus, safe accessibility to these essential services would benefit several at-risk groups, including young women and all caregiving parents with young children (< 5 years of age) at home, who are experiencing disproportionate psychological distress and possible increases in self-harm during the pandemic (Pierce et al., 2020; Ueda et al., 2021).

### Ongoing recommendations

*Recommendation 4.* The COVID-19 pandemic offers a critical opportunity to invest in and strengthen mental-health systems to achieve a “parity of esteem,” meaning that someone who is mentally ill should have equal access to evidence-based treatment as someone who is physically ill. As this article demonstrates, COVID-19 led to an early increase in psychological distress, and elevated reports of depression persisted for months. However, in a sample of 44,775 U.K. adults surveyed between late March to late April, only 40% of people reporting self-harm and suicidal ideation accessed one or more means of formal mental-health support services during the first month of lockdown (Iob, Steptoe, et al., 2020). Likewise, only 12% of college students in France expressing psychological-distress concerns reported seeking professional help (Wathelet et al., 2020). Given that the current concern and interest in mental health and subjective well-being might fade, we argue that now is the time to invest in mental-health services. These services will ideally be free or heavily subsidized so that they are accessible to all. This would help ease the burden of the pandemic and build resources so that individuals, communities, and nations are better able to handle future stressors.

*Recommendation 5.* Specific mental-health resources and actions should be tailored to the resources available but at the very least should include online cognitive-behavior therapy (eCBT) treatments supplemented by locally trained, although possibly lay, mental-health practitioners. Offering universal recommendations is challenging given the range in resources (i.e., low- vs. high-income countries), as well as cultural and ethnic practices (Diala et al., 2001; Gonzalez et al., 2011). However, several overarching suggestions emerge. First, the physical-distancing requirements to slow the spread of the virus make online mental-health treatments an attractive and viable option. eCBT has been shown to be effective in treating depression, anxiety, and loneliness and should therefore be widely available (Etzelmueller et al., 2020; Masi et al., 2011; Phillips et al., 2019). Second, eCBT sessions should be supplemented by occasional meetings with a therapist or local mental-health practitioner. If therapists and practitioners are scarce, community members could be trained to help with implementation and support, but care should be taken because scaling and one-off training can undermine high-fidelity treatment. Fortunately, early data from Ethiopia, Thailand, parts of India, and the United Kingdom suggest that newly trained practitioners and even peers can be effective in task sharing when supported through community mobilization, strong leadership, awareness, stigma reduction, and support provision, as well as credit and recognition (Shidhaye et al., 2017; Singla et al., 2017; Van Ginneken et al., 2017). Third, future research is needed to continue examining the efficacy of scaled treatment (Eaton et al., 2011), and to identify eCBT resources that are effective across cultures and available in relevant translations.

*Recommendation 6.* Individuals and organizations, including health-care providers, should supplement existing mental-health care with well-being promotion. The literature on positive psychology offers a range of relatively easy, low-cost evidence-based strategies that can be implemented to increase the frequency of positive emotions and well-being (VanderWeele, 2020). Strategies include mindfulness (Campos et al., 2016; Fredrickson et al., 2008; Grossman et al., 2004), gratitude (Davis et al., 2016), practicing kindness or generosity (Aknin et al., 2020; Curry et al., 2018; Dunn et al., 2014), and self-compassion or imagining one’s best possible self (King, 2001; Malouff & Schutte, 2017). These tools may be especially useful during the pandemic because they target both positive and negative emotions, which people report having declined and increased, respectively, through COVID-19 (VanderWeele, 2020; Waters et al., 2021). Thus, these practices could help bolster well-being so that people move from left to right on the mental-health continuum shown in Figure 2. These strategies may be particularly useful because, unlike professional mental-health care, these strategies are typically brief, accessible, convenient, self-administered, and nonstigmatizing. Although meta-analyses and/or the use of large preregistered studies support their efficacy, theorizing suggests that strategies may be more or less effective when considering features of the activity (i.e., its variety or stability), features of the actor (i.e., high vs. low motivation), and person-activity fit (see Lyubomirsky & Layous, 2013).

*Recommendation 7.* Governments and organizations should facilitate access to mental-health care and the promotion of well-being alongside social care. Providing citizens or employees with a list of available treatment options has not proven sufficient. Mental-health and well-being activities should involve promotion and community outreach and be contained within the structure of a citizen’s daily life. For instance, schools, workplaces, and community centers should include courses in positive education and positive psychology (Joyce & Paquin, 2016; Kitchener & Jorm, 2004; LaMontagne et al., 2014). Teachers and workplace managers may be more open to these ideas now while responding to the stressors and adjustments of the pandemic, and effective models are available online (e.g., Action for Happiness). To increase the likelihood that valuable resources reach vulnerable populations that need them most, access to and treatment with evidence-based mental-health services should be embedded within existing systems, such as social services, welfare, poverty alleviation, and social-development programs (Patel & Saxena, 2019). Such efforts to build on existing programs and commitments (e.g., universal health coverage, other priority programs) would help to *build back better* and implement a whole government approach to the pandemic that affects all dimensions of our lives. A roadmap to strengthen global mental-health systems to tackle the impact of the COVID-19 pandemic has been proposed and needs to be urgently implemented (Maulik et al., 2020).

## Conclusion

COVID-19 poses one of the largest collective challenges of our lifetime. Although efforts to contain and defeat the virus have understandably been prioritized, mental health should not be ignored during the pandemic or afterward. The impact of the COVID-19 pandemic will likely extend into the future through secondary effects on employment levels, poverty, social inequality, and more (Banks et al., 2021).

Widespread vaccination and the return of prepandemic life is unlikely to be immediate or fully address the mental-health patterns reported here. In fact, we recommend *increasing* attention to mental health over the next few years to prevent widening the gap between mental and physical health care, which could occur for at least two reasons. First, large-scale vaccination will require substantial investment in physical health care. Adding this to the need to reinstate routine physical care will involve significant human, economic, and coordination resources. Second, with physical safety improving, policymakers and the public may assume that most people are prepared to return to a prepandemic routine without attending to the strains on mental health documented here. Thus, we encourage researchers and policymakers to continue monitoring and supporting mental health beyond virus containment and vaccination.

As noted in the introduction, most of the large-scale evidence summarized in this article is drawn primarily from WEIRD nations (Henrich et al., 2010). Although these data offer valuable early insight into how various facets of mental health and well-being are faring during the COVID-19 pandemic in the locations surveyed, nations varied widely in their initial response to the pandemic (Hale et al., 2021). This reality requires careful consideration when trying to understand mental-health responses in lower- and middle-income countries (Kola et al., 2021) as well as global trends. Thus, this limitation raises important opportunities for future research.

A large body of research documents the far-reaching pain caused by mental illness (Layard & Clark, 2015) and, conversely, the numerous benefits of subjective well-being (Lyubomirsky et al., 2005). Thus, subjective well-being measurement and considerations should guide policy, both during the pandemic (e.g., when deciding when to impose and release government lockdowns; De Neve et al., 2020) and beyond (Diener et al., 2009; Diener & Seligman, 2004; Helliwell, 2021; Oishi & Diener, 2014; Sachs, 2019). This refocus should help in several ways. First, it could help to slow the spread of the virus. Recent findings suggest that happier people have stronger immune systems (Diener et al., 2017) and are more likely to comply with public-health measures, such as staying home and maintaining physical distance (Krekel et al., 2020). Indeed, recent evidence indicates that people with greater psychological distress are more likely to have missed or delayed vaccinations during the COVID-19 pandemic (Shapiro & McDonald, 2020). Second, supporting happiness may target other undesirable outcomes, such as “prebunking” conspiracy theories and misinformation (Cichocka, 2020). Finally, a greater focus on subjective well-being would bring the personal experience of citizens to center stage, necessitating ongoing and greater support to help people live fuller, more enjoyable, and connected lives.

## Transparency

*Action Editor:* Joshua Hicks

*Editor:* Laura A. King
